# Unmet need for and impact of adopting immunobiological drugs for moderate to severe plaque psoriasis in a pediatric population

**DOI:** 10.31744/einstein_journal/2024GS0413

**Published:** 2024-05-20

**Authors:** Ana Clara Maia Palhano, Ninna Medeiros Gutierrez, Nicolas Sousa Vecchio dos Santos, Rita Narikawa, André Ballalai, Dimitri Luz Felipe da Silva

**Affiliations:** 1 Dermatology Department Universidade de Santo Amaro São Paulo SP Brazil Dermatology Department, Universidade de Santo Amaro, São Paulo, SP, Brazil.; 2 Faculdade Israelita de Ciências da Saúde Albert Einstein Hospital Israelita Albert Einstein São Paulo SP Brazil Faculdade Israelita de Ciências da Saúde Albert Einstein, Hospital Israelita Albert Einstein, São Paulo, SP, Brazil.

**Keywords:** Psoriasis, Secukinumab, Ustekinumab, Etanercept, Evaluation studies as topic, Health surveillance, Public Health, Therapeutics, Child

## Abstract

**Objective:**

Therefore, this study aimed to evaluate the impact of secukinumab and ustekinumab against moderate-to-severe plaque psoriasis in a Brazilian pediatric population with access to public healthcare.

**Methods:**

A survey of immunobiological treatments registered for use against pediatric psoriasis at the National Health Surveillance Agency was conducted. These treatments were compared to the list available in the same treatment category in the public health system through the Clinical Protocol and Therapeutic Guidelines for psoriasis. A quantitative analysis of the data of patients treated with immunobiological drugs the previous year in accordance with the Clinical Protocol and Therapeutic Guidelines was performed using data available in the DATASUS portal.

**Results:**

The public budget impact scenarios analyzed were comparable to the investment already planned for acquiring the only available drug option.

**Conclusion:**

The incorporation of two therapeutic options in the Clinical Protocol and Therapeutic Guidelines list for moderate-to-severe pediatric psoriasis was feasible in a horizon of 5 years compared to the investment into the single option available to pediatric patients. These findings can facilitate the local analysis of budgetary impact and discussions on the feasibility of this therapeutic incorporation at the state level.

## In Brief

Palhano et al. demonstrate the feasibility of incorporating secukinumab and ustekinumab into the Clinical Protocol and Therapeutic Guidelines for moderate to severe psoriasis in pediatric patients.

## Highlights

Incorporation of secukinumab and ustekinumab was economically feasible.

These drugs are options for those who do not respond to or have contraindications to etanercept.

## Graphical abstract

**Figure:**
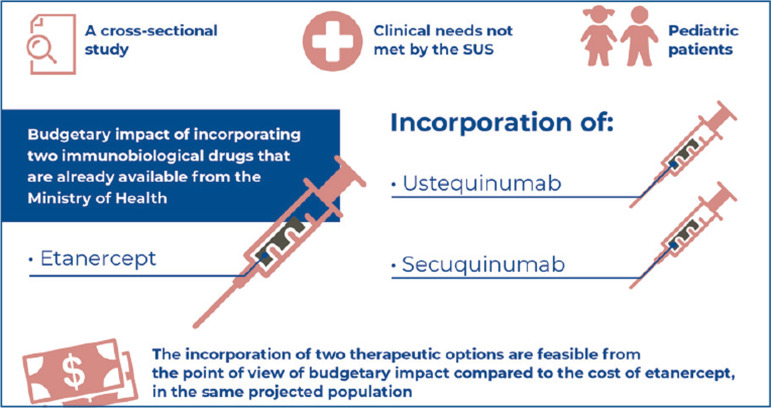

## INTRODUCTION

Pediatric psoriasis substantially impacts patient quality of life and that of their caregivers. In addition, the potential complications of this disease necessitate earlier diagnosis and treatment.^(1,2)^Beyond topical and phototherapy treatments, there are four options for systemic therapy. Of these, etanercept is the only immunobiological drug. The pharmacoeconomic and economic impacts of incorporating health technologies remain largely understudied in Brazil. Moreover, the incorporation process is considerably slow, largely owing to a lack of data and academic interest in the topic. Most studies in these fields are conducted by the National Commission for the Incorporation of Health Technologies (CONITEC - *Comissão Nacional de Incorporação de Tecnologias*) within the scope of the Public Health System (SUS – *Sistema Único de Saúde*), in addition to those conducted or sponsored by the technology manufacturers.^(2,3)^

The regimen and therapeutic options proposed in the Clinical Protocol and Therapeutic Guidelines (CPTG) for psoriasis demonstrate a clear gap in therapeutic options between adult and pediatric patients. The efficacy and safety of secukinumab and ustekinumab demonstrated by the National Health Surveillance Agency (ANVISA - *Agência Nacional de Vigilância Sanitária*) in this population is supported by recent studies.^(4)^ Therefore, a budgetary impact analysis is required to estimate the investment needed for the SUS to integrate a regimen encompassing secukinumab and ustekinumab, in addition to etanercept, into the CPTG for psoriasis. Furthermore, this analysis may demonstrate the investment required to facilitate treatment access through the Specialized Component of the Pharmaceutical Assistance (CEAF - *Componente Especializado da Assistência Farmacêutica*), which is responsible for chronic, complex, and high-budget treatments within the SUS.

## OBJECTIVE

This study aimed to evaluate the impact of secukinumab and ustekinumab against moderate-to-severe plaque psoriasis in a Brazilian pediatric population with access to public healthcare.

## METHODS

This cross-sectional study analyzed the unmet clinical needs of patients, specifically in the treatment of moderate to severe psoriasis in pediatric patients. This study further evaluated the budgetary impact of incorporating two additional immunobiological drugs that are already available from the Ministry of Health.

A data survey of the immunobiological treatments currently registered with the ANVISA for this indication was conducted and compared with the SUS list available in the same category of treatment through the CPTG for psoriasis.

The number of patients whose treatment was in accordance with the current CPTG in the previous year was determined using the data in the DATASUS portal (http://tabnet.datasus.gov.br/cgi/tabcgi.exe?sia/cnv/qauf.def). Information related to treatments already provided for the International Classification of Diseases (ICD) within pediatric indications was selected from this database. A survey of the maximum price for sale to the Government (PMVG - *Preço Máximo de Venda ao Governo*), which is available in the Medicines Market Regulation Chamber (CMED - *Câmara de Regulação do Mercado de Medicamentos*), was conducted. In addition, the latest values for the acquisition of etanercept, secukinumab, and ustekinumab in government contracts were obtained. Two budget impact scenarios were subsequently evaluated and compared to investigate the potential incorporation of the three therapeutic options over a period of 5 years. To construct this scenario, first, an analysis was made of the projection of the Brazilian population between 5 and 19 years of age, available through the population and death projection tables in the Brazilian Institute of Geography and Statistics (IBGE - *Instituto Brasileiro de Geografia e Estatística*) database.

As there are no studies on the incidence or prevalence of moderate-to-severe plaque psoriasis in pediatric patients in Brazil, an inference of 0.05% was made based on the correlation of data between the Brazilian adult population and North American adult and child populations.^(5-7)^

Etanercept is the only immunobiological drug available through CPTG for pediatric patients. Therefore, this study identified pediatric patients with moderate to severe psoriasis who had access to treatment through SUS and no response to other available synthetic treatments. Owing to these characteristics, the patients were potentially eligible for the eventual incorporation of other immunobiologicals into their regimens. The number of active patients using etanercept with the ICDs recommended by the CPTG for psoriasis between September 2020 and August 2021 was estimated.

After the budget impact projection, the clinical and humanistic impacts of these scenarios were evaluated. Finally, recommendations that consider the unmet needs of pediatric patients with psoriasis and the budget impact for the Unified Health System were made.

## RESULTS

The evaluation period was 5 years (2022–2026), as recommended by the Brazilian Budget Impact Assessment Guidelines. First, the projection of the Brazilian population between 5 and 19 years of age was analyzed based on data available through the population and death projections from the database of the IBGE.^(4)^

The effectiveness of the synthetic drug treatments was determined based on the data described in table 1. The CPTG for psoriasis recommend methotrexate as the first synthetic drug based on the described failure rates. In case of failure, the patient would switch to acitretin and finally to cyclosporine. Patients who did not achieve a Psoriasis Area Severity Index (PASI) 75 response to cyclosporine were considered candidates for biologic therapy.^(8)^

**Table 1:** Proposed technologies

Medication	Ustekinumab		Secukinumab	
Commercial name	Stelara^®^		Cosentyx™	
Manufacturer/Importer	Janssen-Cilag Farmaceutic Ltda.		Novartis Pharma Stein AG	
Approved use in Brazil	Psoriatic arthritis, plaque psoriasis, Crohn’s disease, ulcerative colitis		Psoriatic arthritis, plaque psoriasis, ankylosing spondylitis	
Proposed indication	Moderate to severe plaque psoriasis for patients 6 years and older		Moderate to severe plaque psoriasis for patients 6 years and older	
Commercial presentation	45mg/0.5mL injectable solution in a pack of one vial or pre-filled syringe. 90mg/1.0mL injectable solution in a pack of one pre-filled syringe		Pen filled with 75mg/0.5mL of injectable solution. Pen filled with 150mg/1mL of injectable solution	
Route of administration	Subcutaneous		Subcutaneous	
Posology	According to weight at weeks 0 and 4, and then every 12 weeks		According to weight at weeks 0, 1, 2, 3 and 4 and then every 4 weeks	
	Body weight	Recommended Dose	Body weight	Recommended Dose
	<60kg	0.75mg/kg	<25kg	75mg
	≥60kg to ≤100kg	45mg	25kg to <50kg	
	>100kg	90mg	≥50kg	150mg to 300mg
Expected outcomes	PASI 75 at week 12		PASI 75 at week 12	
Treatment time	There are no studies supporting discontinuation by the time of treatment		There are no studies supporting discontinuation by the time of treatment	
Price (PMVG 18% - CMED 2021)	45 mg/0.5 mL of injectable solution (ampoule-bottle)	R$ 10.883,13	75mg/0.5mL of injectable solution (applicator pen)	R$ 1.454,90
	90mg/1.0mL of injectable solution (ampoule-bottle)	R$ 21.766,21	150mg/1mL of injectable solution (applicator pen)	R$ 2.909,80

Source: Agência Nacional de Vigilância Sanitária (ANVISA). Lista de preços máximos de medicamentos por princípio ativo, para compras públicas. Brasília (DF): ANVISA; 2021 [citado 2022 Out 10]. Disponível em: https://www.gov.br/anvisa/pt-br/assuntos/medicamentos/cmed/precos^(21)^

PMVG: *Preço Máximo de Venda ao Governo*; PASI: Psoriasis Area Severity Index.

The growth projection of eligible patients in 5 years considers the growth calculation of the previous 12 months of available data from active patients in DATASUS (September 2020–August 2021). The increase in new patients receiving etanercept in the first 12 months immediately following incorporation (September 2019 to September 2020) represents a pent-up demand from patients due to lack of access, which does not correspond to reality. Therefore, these data would not be useful for this analysis.

The treatment cost for each biological drug was estimated from the PMVG, which is the drug market standard for bidding processes, with an 18% increase in the tax on operations related to the circulation of goods. The presentations and number of doses considered were the same as those recommended in the package inserts and registered with ANVISA (Table 1). For fractioned doses of mg/weight, we assumed the use of one vial/syringe per patient and disposal of the residual content. The total treatment cost was estimated for the first year (induction year) and for the subsequent year (maintenance year). The costs considered in the analysis included only those for purchasing drugs from the SUS (Table 2).

**Table 2:** Costs per treatment and total costs per treatment/year

Treatment	Indication/weight	Dose	Presentation	PMVG 18% (R$)	Year 1 cost	Year 2 cost	Total cost (year 1 + year 2)
Etanercept	<62.5kg	0.8mg/kg	25mg injectable solution in one pre-filled 1mL syringe	-	-	-	-
	≥62.5kg	50mg	50mg injectable solution in one pre-filled 1mL syringe	2.307,03	0,00	110.737,44	110.737,44
Secukinumab	<25kg	75mg	75mg injectable solution in one pre-filled 0.5mL syringe	1.454,90	24.733,30	17.458,80	42.192,10
	≥25kg and <50kg						
	≥50kg	150mg	150mg injectable solution in one pre-filled 1mL syringe	2.909,80	49.466,60	34.917,60	84.384,20
	≥50kg	300mg	150mg injectable solution in two pre-filled 1mL syringes	5.819,60	98.933,20	69.835,20	168.768,40
Ustekinumab	<60kg	0.75mg/kg	45mg injectable solution in a 0.5mL vial	10.883,14	97.948,26	43.532,56	141.480,82
	≥60kg and <100kg	45mg					

Source: Agência Nacional de Vigilância Sanitária (ANVISA). Lista de preços máximos de medicamentos por princípio ativo, para compras públicas. Brasília (DF): ANVISA; 2021 [citado 2022 Out 10]. Disponível em: https://www.gov.br/anvisa/pt-br/assuntos/medicamentos/cmed/precos^(21)^

^PMVG: *Preço Máximo de Venda ao Governo*.^

In Scenario 1, the adoption of equal proportions of patients using the three drugs was considered because they were equally available. Efficacy and safety studies of the three drugs, as well as recommendations from international agencies, demonstrated their comparative effectiveness and safety for pediatric patients with psoriasis. Additionally, the proportion of patients in the pivotal studies on this indication for secukinumab and ustekinumab was used to calculate the dosage by weight and the cost of treatment. In total, 47.5%, 26.2%, and 26.2% of patients weighing ≤50kg, ≥50kg, and ≥50 kg received 75mg, 150mg, and 300mg of secukinumab, respectively. In contrast, 91% and 9% of patients weighing <60kg and ≥60kg received up to 45mg and 45mg of ustekinumab, respectively. No population in this indication weighed >100kg. For etanercept, the same premise was used as that in the CONITEC Recommendation Report No. 385 (http://antigo-conitec.saude.gov.br/images/Relatorios/2018/Relatorio_Biologicos_Psoriase.pdf), adopting an average weight of 70kg per patient (Table 3 and Table 4).

**Table 3:** Annual treatment cost (R$) - PMVG 18%

Drug	PMVG 18% (unity)	Year 1 induction cost	Year 2 maintenance cost	Total (year 1 + year 2)
Etanercept	2.307,03	110.737,44	110.737,44	221.474,88
Secukinumab	2.909,80	49.466,60	34.917,60	84.384,20
Ustekinumab	10.883,14	97.948,26	43.532,56	141.480,82

Source: Agência Nacional de Vigilância Sanitária (ANVISA). Lista de preços máximos de medicamentos por princípio ativo, para compras públicas. Brasília (DF): ANVISA; 2021 [citado 2022 Out 10]. Disponível em: https://www.gov.br/anvisa/pt-br/assuntos/medicamentos/cmed/precos^(21)^

^PMVG: *Preço Máximo de Venda ao Governo*.^

**Table 4:** Budget Impact (R$) - Scenario 1 (PMVG 18%)

Treatment period	Etanercept 33%	Secukinumab 33%	Ustekinumab 33.33%
2022	203.741.509,40	6.525.523,95	8.302.365,27
2023	276.600.592,84	7.326.060,74	10.514.608,78
2024	349.419.687,76	9.134.460,80	11.036.999,02
2025	422.278.771,20	9.980.272,38	13.152.740,38
2026	495.097.866,12	12.751.263,92	15.442.611,82
Total	1.747.138.427,32	45.717.581,79	58.449.325,27

^PMVG: *Preço Máximo de Venda ao Governo*.^

In this scenario, treatment adoption was considered from January 2022 to calculate secukinumab and ustekinumab induction. Etanercept had no induction, with the cost in Year 1 being equivalent to the maintenance cost. Moreover, the response rate to each drug was not considered. Therefore, all patients were treated with the evaluated drug until the end of the proposed model.

Although the PMVG is the reference value for public purchases, manufacturers often present acquisition cost proposals below the PMVG when there is a possibility of incorporation. This facilitates a lower total budget impact and greater feasibility and possibility of incorporation, as can be seen in CONITEC Recommendation Report No. 385. Therefore, the costs of the same presentations were considered based on the last sale made to the Ministry of Health^(9)^to serve the adult population for Scenario 2 (Tables 5 and 6).

**Table 5:** Prices of previous and projected sales to the government (R$)

Year	Total eligible population	Etanercept total cost
2022	5.095	42.588.901,20
2023	6.917	57.818.926,32
2024	8.738	73.040.592,48
2025	10.560	88.270.617,60
2026	12.381	103.492.283,76
Total	43.691	365.211.321,36

**Table 6:** Budget Impact (R$) - Scenario 2

Treatment period	Etanercept 33.33%	Secukinumab 33.33%	Ustekinumab 33.33%
2022	14.196.300,40	1.323.701,17	3.023.716,89
2023	19.272.975,44	1.486.089,68	3.829.414,77
2024	24.346.864,16	1.852.923,46	4.631.675,66
2025	29.423.539,20	2.024.496,15	5.351.226,95
2026	34.497.427,92	5.065.584,37	6.236.197,43
Total	121.737.107,12	11.752.794,83	23.072.231,70

The 75 mg presentation has not been included in the CPTG yet, and no contract was found to supply this presentation to the Ministry of Health. Thus, the same proportion as that of cost per dose in the PMVG table was assumed for the calculation of Scenario 2, with the 75mg presentation being half the cost of the 150mg presentation. The proportions of treatment adoption and dosage regimens followed the same assumptions as those in Scenario 1 (Table 7).

**Table 7:** Projected total budget impact (R$) compared to previous government sales

Year	Etanercept 100%	Etanercept, Secukinumab, and Ustekinumab
2022	42.588.901,20	18.543.718,46
2023	57.818.926,32	24.588.479,89
2024	73.040.592,48	30.831.463,28
2025	88.270.617,60	36.799.262,30
2026	103.492.283,76	45.799.209,72
Total	365.211.321,36	156.562.133,65

## DISCUSSION

Psoriasis is a chronic inflammatory disease characterized by erythematous scaly lesions and nail and joint manifestations. This disease can affect up to 8.5% and 2.1% of the adult and pediatric populations, respectively.^(1,2)^It is commonly associated with metabolic syndrome, with 26% of patients with psoriasis developing systemic arterial hypertension and 11.7% developing diabetes mellitus. Moreover, this disease is an independent risk factor for acute myocardial infarction. The cumulative impact on the life course of individuals with psoriasis, as well as their quality of life, work productivity, and interpersonal relationships has been demonstrated. Approximately 15.4% and 16% of patients with psoriasis develop anxiety and depression, respectively, with up to 2.9% attempting suicide.^(10,11)^

The prevalence of psoriasis in pediatric populations is approximately 0.1–1.37%, occurring preferentially in older children, with the percentage of 0.12 at the age of 1 year increasing to 1.24 at 18 years.^(12)^ Studies have also demonstrated an increase in the diagnosis of pediatric psoriasis over time (29.6 per 100,000 children in 1974 and 62.7 per 100,000 children in 1999), which may be due to an increase in the prevalence of childhood obesity, one of the main risk factors in this population.^(13)^

Psoriasis often affects different locations in pediatric patients compared to adults. These areas include the face, scalp, and diaper areas. As is typical of psoriatic disease, comorbidities acquire greater clinical concern, given the increased possibility of therapeutic failure and drug exhaustion in patients with chronic or prolonged comorbidities. Of all comorbidities, obesity, cardiovascular diseases, psoriatic arthritis, and psychosocial disorders are potentiated by early onset and are difficult to control.^(14)^

Calcipotriol, a vitamin D3 analog and topical corticosteroid, is one of the most commonly used topical drugs. Furthermore, PUVA and narrowband UVB phototherapy are widely used to treat psoriasis. Vitamin A-derived retinoids: isotretinoin and acitretin are systemic medications available which help manage the keratinization component of the disease. Similarly, methotrexate is an antiproliferative and inhibitor of folic acid metabolism that acts as a potent anti-inflammatory agent and promotes an important improvement in joint disease.^(15-17)^

The Brazilian Consensus on Psoriasis, updated in 2020, classifies moderate to severe psoriasis according to the following criteria: Psoriasis Area Severity Index (PASI), Dermatology Life Quality Index, and/or body surface area >10, as well as candidacy for systemic therapy in both children and adults. Initial treatment with phototherapy is suggested in the Brazilian Consensus, which focuses on the adult population. However, conventional systemic therapies such as methotrexate and acitretin are used if this is not available in the service or city of the patients. In the event of failure, loss of response, or the inapplicability of these therapies, biological therapy is recommended.^(18)^

The SUS divides pharmaceutical care into main components to assess the needs and availability of medication. The CEAF is responsible for investments in medicines, largely for the incorporation of immunobiological treatments for specialized chronic and rare diseases. This specialized component of the SUS makes recommendations based on the CPTG. These guidelines rely on the epidemiological data of a particular disease or condition to define the parameters and criteria for carrying out the diagnosis, treatment, and monitoring within the scope of the SUS and its available inputs and services. The evaluation of technologies to be included in these protocols, with consequent funding and availability to the population via the SUS care network, is carried out by CONITEC, which is responsible for technical evaluation (safety, effectiveness, economic analysis, and budgetary impact) and issuance of positive or negative recommendations. Finally, the Secretariat of Science, Technology, Innovation, and Strategic Inputs (SCTIE - *Secretaria de Ciência, Tecnologia, Inovação e Insumos Estratégicos*) defines incorporation or non-incorporation based on the assessment of budgetary impact and civil society manifestations in public consultations.

The pediatric psoriasis scenario in Brazil includes the incorporation of six therapies funded by the SUS: phototherapy, calcipotriol cream, methotrexate, acitretin, cyclosporine, and etanercept. Phototherapy is not available to most of the population; calcipotriol is used as a complementary therapy or as monotherapy in isolated lesions; methotrexate has potentially adverse events, and its prolonged use can lead to cumulative damage to liver function; and cyclosporine is used only for rescue situations, and its chronic use is not recommended. Therefore, acitretin is currently used as a classic drug and etanercept is a biological option for moderate to severe plaque psoriasis in the pediatric population.^(18-21)^ However, there are no studies on failure of the therapeutic response to etanercept or potential switches to other immunobiological drugs in the pediatric population. Consequently, pediatric patients have no access to a second therapeutic option if they present with contraindications or therapeutic failure of etanercept. This represents a major limitation in the CPTG attempt to achieve the proposed goals for the treatment of moderate to severe psoriasis.

Secukinumab and ustekinumab have been incorporated into the CPTG for adult patients and are recommended for pediatric populations with moderate to severe psoriasis in the package insert. The National Institute for Health and Care Excellence (NICE) of the British health system has recommended the use of adalimumab, etanercept, and ustekinumab in pediatric patients since 2017.^(22)^ Adalimumab and etanercept are recommended for patients older than 4 and 6 years, respectively. In contrast, ustekinumab is recommended for patients who are 12 years of age or older. All three drugs are indicated for severe forms of the disease and for non-responders with intolerance or contraindication to systemic treatments such as cyclosporine, methotrexate, or phototherapy.^(22)^ However, secukinumab had not yet been recommended for the pediatric population in the package insert in 2017.

Children have a longer exposure to the disease. Thus, they are more likely to develop psoriatic arthritis and other comorbidities, leading to low productivity at work, absenteeism, hospital admissions, and high costs of outpatient consultations and psychiatric treatments in their adult lives. Each patient can present a particular response to a singular therapy. As such, there may also be drug refractoriness in addition to intolerance. Therefore, these aspects should be considered from both a public health perspective and the perspective of offering varied and feasible treatments for this population.

Comparison of Scenarios 1 and 2 clearly demonstrates that treatment combinations based on the PMVG can be more feasible and have substantially greater budgetary impact compared to those based on negotiated price proposals, especially those agreed upon in a centralized purchase by the Ministry of Health. Etanercept is the only treatment incorporated for the pediatric population, possibly owing to considerable differences between the PMVG price (R$ 2,307.03) and that of the last government purchase (R$ 160.75). This suggests that the offer of negotiated price proposals is considered more feasible for financing by the SUS and for the manufacturers; it is assumed that negotiated price proposals are advantageous and do not present losses for either stakeholder.

The two budget impact scenarios demonstrate that the combination of secukinumab and ustekinumab with etanercept treatment is more economically feasible than etanercept alone in the same projected population that is eligible for the presently incorporated treatment. This result is valid for the proportional adoption of the three treatments (Table 6) as well as when observing the budget impact projection of etanercept in 5 years without the current CPTG update and without the incorporation of new therapeutic options (Table 7).

It was not the objective of this study to evaluate the feasibility of incorporation by the SCTIE. This was because the SCTIE relies on its own studies of the available budget, articulation, and accommodation of numerous competing health needs and demands; inter-manager agreements; and other deliberations within the scope of the Ministry of Health, states, and municipalities. However, our analysis confirms that the cost of incorporating two new therapeutic options that are safe and effective, considering the premises set out in this study, may not result in additional costs to what will already be invested based on the current CPTG in 5 years. Therefore, it is economically feasible.

Despite being a reference for public purchases, the PMVG appear unrealistic for evaluating incorporations of immunobiologicals for psoriasis treatment within the scope of the SUS. Thus, it is reasonable to infer that the incorporation of etanercept at the PMVG price would not have received a favorable recommendation from CONITEC Recommendation Report No. 385 ((http://antigo-conitec.saude.gov.br/images/Relatorios/2018/Relatorio_Biologicos_Psoriase.pdf). Offering price proposals and special negotiations for incorporation is an accepted practice for budget impact analysis, and has been cited in the NICE recommendation report. In compliance with Law No. 8,666 of June 21, 1993 (https://www.gov.br/saude/pt-br/acesso-a-informacao/banco-de-precos/legislacao/lei-no-8-666-de-21-de-junho-de-1993.pdf/view), which regulates public purchases, these negotiations were considered and encouraged in this study, as they have a direct and considerable effect on budgetary impact analysis and may directly impact the feasibility of incorporation.

The objective of this study was not to evaluate the impact of the judicialization of health on the treatment of psoriasis in pediatric patients. This topic has been widely discussed because of its impact on the health budget, especially at the state level. However, the data are difficult to access and are not part of the DATASUS scope. They are not standardized and there are often no details by presentation or indication. However, considering that the PMVG establishes a mandatory minimum discount and maximum price to meet legal demands, our findings can help support the local analysis of budgetary impacts and discussions on the feasibility of incorporation at the state level. Although the analysis presented here considers the reality, needs, and assumptions of federal incorporation, the comparison of budget impact with the PMVG price and a special price proposal for incorporation can help managers outline local negotiation strategies with suppliers.

Finally, the theoretical framework presented and budget impact results analyzed in the present study provide a basis for qualifying secukinumab and ustekinumab as feasible options for patients who do not respond to or are contraindicated for use with current standard synthetic systemic therapy. Both drugs have been proven to be safe and effective in the treatment of pediatric patients with moderate to severe plaque psoriasis. There is no doubt about the high cost that the incorporation of these technologies would present to the health system. However, the economic and humanistic impact of untreated moderate to severe plaque psoriasis on pediatric patients and their caregivers, as well as the indirect cost to the health system, are yet to be assessed. Thus, the results presented here may encourage future analyses and studies aimed at better managing the resources used in the treatment of psoriasis. This would facilitate the use of these resources for the benefit of the population and public health system.

## CONCLUSION

The two budget impact scenarios analyzed in this study demonstrate that the incorporation of secukinumab and ustekinumab with etanercept is economically feasible compared to etanercept alone in the same projected population that is eligible for the presently incorporated treatment.

The development of new drugs and indication of new age groups in pre-existing medications warrant the review of the Clinical Protocol and Therapeutic Guidelines for moderate to severe psoriasis to include the pediatric population. Alternatively, new Clinical Protocol and Therapeutic Guidelines for moderate-to-severe psoriasis that are specific to the pediatric population must be developed.
